# *Kocuria kristinae *infection associated with acute cholecystitis

**DOI:** 10.1186/1471-2334-5-60

**Published:** 2005-07-19

**Authors:** Edmond SK Ma, Chris LP Wong, Kristi TW Lai, Edmond CH Chan, WC Yam, Angus CW Chan

**Affiliations:** 1 Division of Clinical Pathology, Department of Pathology, Hong Kong Sanatorium & Hospital, Hong Kong; 2 Private Practitioner in Family Medicine, Hong Kong; 3 Department of Microbiology, The University of Hong Kong, Hong Kong; 4 Minimal Invasive & Endoscopic Surgery Centre, Hong Kong Sanatorium & Hospital, Hong Kong

## Abstract

**Background:**

*Kocuria*, previously classified into the genus of *Micrococcus*, is commonly found on human skin. Two species, *K. rosea *and *K. kristinae*, are etiologically associated with catheter-related bacteremia.

**Case presentation:**

We describe the first case of *K. kristinae *infection associated with acute cholecystitis. The microorganism was isolated from the bile of a 56-year old Chinese man who underwent laparoscopic cholecystectomy. He developed post-operative fever that resolved readily after levofloxacin treatment.

**Conclusion:**

Our report of *K. kristinae *infection associated with acute cholecystitis expands the clinical spectrum of infections caused by this group of bacteria. With increasing number of recent reports describing the association between *Kocuria spp. *and infectious diseases, the significance of their isolation from clinical specimens cannot be underestimated. A complete picture of infections related to *Kocuria spp. *will have to await the documentation of more clinical cases.

## Background

*Kocuria *is a member of the *Micrococcaceae *family and consists of nine species. It was previously classified into the genus of *Micrococcus*, but was dissected from *Micrococcus *based on phylogenetic and chemotaxonomic analysis [1]. The organism is widespread in nature and is frequently found as normal skin flora in humans and other mammals. Documented cases of infections due to *Kocuria spp. *are limited. The type species *K. rosea *has been reported to cause catheter-related bacteremia [2]. Another member of the genus, *K. kristinae *(previously known as *Micrococcus kristinae*), was first described in 1974 [3]. This organism is an aerobic, gram-positive coccus occurring in tetrads, and the majority of strains are non-pathogenic. Clinically similar to *K. rosea*, a single case of catheter-related bacteremia due to *K. kristinae *has been reported in a patient with ovarian cancer [4]. Here we report the first case of *K. kristinae *isolated from bile in a patient with acute cholecystitis.

## Case presentation

A 56-year old Chinese man, who had a known history of asymptomatic gallstones, presented with right upper quadrant abdominal pain for five days associated with fever. Laboratory investigations showed neutrophilia, but the liver function test was normal. Ultrasound examination of the abdomen revealed distended gallbladder associated with multiple gallstones, prominent intrahepatic ducts and enlarged lymph nodes at the porta hepatitis region. Laparoscopic cholecystectomy performed for a diagnosis of acute cholecystitis showed distended and gross thickened gallbladder and omental adhesions. The bile was turbid and two stones were found impacted at the Hartmann's pouch. The cystic duct was normal. The patient developed post-operative fever and intravenous levofloxacin at a dosage of 500 mg daily was started as empirical treatment. Bile culture subsequently yielded a pure growth of *K. kristinae *(see microbiology diagnosis). Fever resolved readily after levofloxacin therapy, which was continued orally at the same dosage for a total duration of 14 days. He made an uneventful recovery.

## Microbiological diagnosis

Culture of bile from gall bladder was performed with sheep blood agar, MacConkey agar and chocolate agar. The plates were incubated at 35°C for 48 hours. Anaerobic culture was performed using Schaedler blood agar and incubated at 35°C for 48 hours. Gram-positive cocci arranged in tetrads were isolated from pale cream colonies after two days incubation. The organism was non-hemolytic, catalase positive, coagulase negative and non-motile. Identification was performed using Biomerieux ID32 Staph ATB system and BD Phoenix PMC/ID-13 system. The isolate was identified as *Kocuria kristinae *with a probability of identification of 99.9 % and confidence value of 99% for the ATB system and Phoenix system respectively. Identification of the isolate was confirmed using 16S rRNA sequencing (MicroSeq™, Applied Biosystems, USA), as misidentification of coagulase-negative staphylococci as *Kocuria *species has been described [5]. Analysis of nucleotide sequence with BLAST programs showed 100% DNA sequence homology with *K. kristinae*. Antibiotic sensitivity test was performed using the disc diffusion method according to Clinical and Laboratory Standards Institute (formerly NCCLS) guidelines for *Staphylococcus*. The isolate was sensitive to penicillin, cloxacillin, erythromycin, clindamycin, linezolid, trimethoprim/sulfamethoxazole, vancomycin and levofloxacin.

## Discussion

Members of the genus *Micrococcus *are found as normal flora of the skin and mucosa. Infections related to *Micrococcus spp. *are uncommon but are recognized, especially in immunocompromised patients with underlying diseases. The organism *M. luteus *has been described as the causative agent in meningitis [6], intracranial abscess [7], arthritis [8], pneumonia [9] and catheter-related sepsis in patients undergoing hemodialysis [10] or leukaemia treatment [11]. Other infections associated with *Micrococcus *and related organisms include continuous ambulatory dialysis peritonitis [12], endocarditis [13] and infection of cerebrospinal fluid shunts [14]. More recently, *Micrococcus spp. *is implicated in central venous catheter infection in patients with pulmonary hypertension receiving continuous epoprostenol infusion [15,16].

*Kocuria *is previously classified as *Micrococcus *and, being inhabitants of the skin, it is not surprising that *K. rosea *and *K. kristinae *have been incriminated as pathogens causing catheter-related bacteremia [2,4]. Misidentification of coagulase negative staphylococcus as *Kocuria *using standard biochemical analysis is not uncommon due to phenotypic variability [5]. The utilization of genotypic assay such as 16s rRNA is required to confirm species identity as in the present case may be required, particularly for unusual clinical scenarios. The *K. kristinae *organism isolated in our patient was sensitive to most of the commonly used antibiotics. A report in the literature on 219 strains of *Kocuria *and *Micrococcus *shows that most strains are sensitive to doxycycline, cetriaxone, cefuroxime, amikacin, and amoxicillin with clavulanic acid, but most are resistant to ampicillin and erythromycin [17]. The duration of therapy in general depends on site and severity of infection. If bacteremia is present or likely, duration of 10 – 14 days is commonly employed.

Bile cultures are sterile in 25 – 50% of acutely inflamed gallbladders. Bacterial infection in acute cholecystitis is usually a secondary event, and is most commonly due to enteric bacteria. A recent study from the Netherlands on microbes isolated from bile after cholecystectomy [18] showed a predominance of *Escherichia coli*, followed by *Klebsiella spp. *and *Streptococcus spp. *Significantly, two studies on infective complications after open [18,19] and laparoscopic cholecystectomy showed no correlation between positive bile culture and post-operative infection. These findings, together with the lower incidence of wound infections after laparoscopic cholecystectomy, would cast doubt on the use of routine antibiotics prophylaxis as recommended for biliary surgery. However, the development of post-operative fever in our patient necessitated the use of empirical antibiotic cover. Levofloxacin used in the present case is a third generation fluoroquinolone with a broad spectrum of antibacterial activity, which has been shown to give adequate serum and gallbladder tissue concentrations in biliary tract surgery [20].

We describe the first case of *K. kristinae *infection associated with acute cholecystitis. Interestingly, a related skin commensal *Staphylococcus aureus *has been recognized as the primary pathogen in unusual cases of acute cholecystitis [21]. *S. aureus *associated acute cholecystitis might be encountered in the clinical setting of bacteremia due to infective endocarditis or nosocomial acquisition in patients with chronic medical conditions [21]. While unfortunately a blood culture was not taken in our patient, the presence of gallstone, good pre-morbid status and prompt resolution of fever after antibiotics would point against a possible endovascular focus of infection.

## Conclusion

Although previously regarded as an innocuous microorganism, there have been a number of recent reports describing the association between *Kocuria spp. *and infectious diseases. The complete clinical spectrum of infections caused by this group of bacteria will be more apparent after the report of more cases. The physician should not therefore underestimate the importance of *K. kristinae *when isolated from clinical specimens.

## Competing interests

The author(s) declare that they have no competing interests.

## Authors' contributions

ESKM, CLPW and KTWL carried out the laboratory studies of the patient. WCY performed the 16s rRNA sequencing. ACWC performed the operation and provided clinical details. ECHC followed up the patient and obtained consent from the patient to publish this case report. ESKM and CLPW drafted the manuscript. All authors read and approved the final manuscript.

## Pre-publication history

The pre-publication history for this paper can be accessed here:


